# A Multi-Omics Approach Reveals New Signatures in Obese Allergic Asthmatic Children

**DOI:** 10.3390/biomedicines8090359

**Published:** 2020-09-18

**Authors:** Mª Amelia Gomez-Llorente, Ana Martínez-Cañavate, Natalia Chueca, Mª de la Cruz Rico, Raquel Romero, Augusto Anguita-Ruiz, Concepción Mª Aguilera, Mercedes Gil-Campos, Maria D Mesa, Bekzod Khakimov, Jose Antonio Morillo, Ángel Gil, José Camacho, Carolina Gomez-Llorente

**Affiliations:** 1 Pediatric Unit, Hospital Materno-Infantil, Ciudad Sanitaria Virgen de las Nieves, 18014 Granada, Spain; mariaa.gomez.sspa@juntadeandalucia.es; 2 Pediatric Allergology Unit, Hospital Materno-Infantil, Ciudad Sanitaria Virgen de las Nieves, 18014 Granada, Spain; anamartinezcanavate@gmail.com; 3 Department of Microbiology, Complejo Hospitalario de Granada, 18012 Granada, Spain; naisses@yahoo.es; 4 ibs.GRANADA, Instituto de Investigación Biosanitaria, 18012 Granada, Spain; augustoanguitaruiz@gmail.com (A.A.-R.); caguiler@ugr.es (C.M.A.); mdmesa@ugr.es (M.D.M.); agil@ugr.es (Á.G.); 5 Redes Temáticas de Investigación Cooperativa RETIC (Red SAMID RD12/0026/0015) Instituo de Salud Carlos III, 28029 Madrid, Spain; mdcrico@ugr.es; 6 Department of Biochemistry and Molecular Biology II, School of Pharmacy, Institute of Nutrition and Food Technology “José Mataix”, Biomedical Research Center, University of Granada, 18160 Granada, Spain; 7 Pediatric Unit, San Cecilio University Hospital, 18012 Granada, Spain; rakelina20@hotmail.com; 8 CIBEROBN (Physiopathology of Obesity and Nutrition CB12/03/30038), Instituto de Salud Carlos III, 28029 Madrid, Spain; mercedes_gil_campos@yahoo.es; 9 Unit of Pediatric Endocrinology, Reina Sofia University Hospital, 14004 Córdoba, Spain; 10 Department of Food Science, University of Copenhagen, Rolighedsvej 26, 1958 Frederiksberg, Denmark; bzo@food.ku.dk; 11 Estación Experimental de Zonas Áridas, Consejo Superior de Investigaciones Cientificas (EEZA-CSIC), Carretera de Sacramento s/n, La Canada de San Urbano, 04120 Almería, Spain; jamorillo.microb@gmail.com; 12 Department of Signal Theory, Networking and Communications, University of Granada, 18071 Granada, Spain; josecamacho@ugr.es

**Keywords:** asthma, gastrointestinal microbiome, metabolomics, multi-omics approach

## Abstract

**Background:** Asthma is a multifactorial condition where patients with identical clinical diagnoses do not have the same clinical history or respond to treatment. This clinical heterogeneity is reflected in the definition of two main endotypes. We aimed to explore the metabolic and microbiota signatures that characterize the clinical allergic asthma phenotype in obese children. **Methods:** We used a multi-omics approach combining clinical data, plasma and fecal inflammatory biomarkers, metagenomics, and metabolomics data in a cohort of allergic asthmatic children. **Results:** We observed that the obese allergic asthmatic phenotype was markedly associated with higher levels of leptin and lower relative proportions of plasma acetate and a member from the *Clostridiales* order. Moreover, allergic children with a worse asthma outcome showed higher levels of large unstained cells, fecal D lactate and D/L lactate ratio, and with a higher relative proportion of plasma creatinine and an unclassified family member from the RF39 order belonging to the Mollicutes class. Otherwise, children with persistent asthma presented lower levels of plasma citrate and dimethylsulfone. **Conclusion:** Our integrative approach shows the molecular heterogeneity of the allergic asthma phenotype while highlighting the use of omics technologies to examine the clinical phenotype at a more holistic level.

## 1. Introduction

Asthma and obesity are two common chronic health problems affecting children [1,2]. Asthma is a heterogenetic chronic disease of the airways characterized by recurrent respiratory symptoms, bronchoreactivity and airway inflammation [1]. Asthma symptoms vary from mild to severe, with sometimes life-threatening exacerbations. Most asthmatic children present a mild or moderate disease form, whereas around 5% suffer from severe symptoms of the disease [3]. Despite the clinic heterogeneity, two main endotypes have been described: T helper 2 (Th2)-high asthma and Th2-low asthma. In the former, we can find three phenotypes: early-onset allergic asthma, late-onset eosinophilic asthma, and aspirin-exacerbated respiratory disease. Early-onset allergic asthma is characterized by a positive allergy skin test and increased serum-specific immunoglobulin (Ig) E [4].

Obesity, defined by an increase in Body Mass Index (BMI), could negatively affect the respiratory and the immune system, increasing the risk of developing asthma. Three distinct asthma phenotypes associated with obesity can be distinguished: early-onset asthma, late-onset asthma and irritant/pollution-associated asthma and obesity [5,6,7]. Early-onset asthma is characterized by a higher prevalence of allergic disease [7]. In a recent meta-analysis performed in the pediatric population, early-onset asthma and wheezing were associated with a higher incidence of childhood obesity. The presence of allergic rhinitis has also been associated with a higher risk of obesity [8]. Although some studies have shown a relationship between early-onset allergic asthma and obesity, other studies have described that in the pediatric population, obesity is more closely related to non-atopic asthma [8,9].

Different mechanisms, apart from the obesity mechanical effect, have been proposed to explain the association between these two diseases. Dyslipidemia and insulin resistance, both of them characteristic of the metabolic syndrome observed in children with obesity [10], have been associated with an impaired lung function [11]. Moreover, obesity is recognized as a chronic low-grade systemic inflammation disorder. The adipose tissue is an endocrine organ, responsible for the secretion of different adipokines. In subjects with obesity, the level of leptin, an inflammatory protein, is increased, while the level of adiponectin, a well-known anti-inflammatory protein, is decreased. One important factor in the development and function of the immune system is the intestinal microbiota [12]. Studies have demonstrated that intestinal dysbiosis is present in obese and asthmatic patients [13,14]. Changes in the taxonomic composition of the intestinal microbiota results in a modification of its fermentation capacity leading to a modification of short-chain fatty acid (SCFA) production that may affect the development of allergic airway disease. On this matter, in mice models, propionate and acetate have been associated with an attenuated allergic airway inflammation [15,16]. During adulthood, obese-asthma patients likely tend to become corticosteroid resistant and often express a severe form of asthma [17,18,19]. A recent study showed that specific immunological and microbiome alterations are associated with obesity and asthma in adult populations [19]. However, our knowledge of pediatric asthma–obesity association is limited and sometimes contradictory [1]. The exact molecular and microbiota characteristics underlying the early-onset allergic asthma phenotype associated with obesity are also very limited.

In the present study, we hypothesized that obese allergic children have a specific molecular signature compared to non-obese asthmatic children. For this, we performed a multi-omics approach by combining multivariate datasets including intestinal microbiota (16S rRNA gene barcoded sequencing), blood and fecal inflammatory biomarkers, blood plasma metabolites (untargeted nuclear magnetic resonance spectroscopy metabolomics and SCFA), and clinical, biochemical, and anthropometrical data. As a secondary outcome, we aim to characterize the persistent asthma phenotype using the same approach.

## 2. Materials and Methods

### 2.1. Study Design and Subjects

A total of 46 allergic asthmatic children (the BIOASMA cohort), compromised by 12 girls and 34 boys, aged 4–13 years, were recruit from the Pediatric Unit of Hospital Virgen de las Nieves and Hospital Universitario San Cecilio (Granada, Spain). The inclusion criteria for the study were as follows: (1) a clinical diagnosis of asthma, defined by the presence of at least three episodes of coughs and wheeze; (2) aged between 1–13 years old; (3) diagnosis of type I allergy based on the Gell–Coombs classification of hypersensitivity reactions. The exclusion criteria were the following: (1) diagnosis of a disease compatible with the clinic’s asthma symptoms, such as cystic fibrosis; (2) preterm birth; (3) malnutrition or the presence of another disease (diabetes mellitus type 2, hepatic or kidney-related disease) (4) use of oral glucocorticoids or other medication that could influence the gastrointestinal microbiome, glucose or lipid metabolisms; and (5) to be on immunotherapy treatment. The use of inhaled corticoids was allowed.

The severity of asthma was determined according to the Spanish Guidelines for Asthma Management (GEMA criteria 4.4, Guía Española para el Manejo del Asma) [20], which was adapted from the GINA recommendations (www.ginasthma.org). In children, two main patterns of asthma can be defined: episodic asthma (occasional or frequent) based on the number of crises that it presents, and persistent asthma (moderate or severe) [20]. Asthma severity was determined by trained pediatrics according to the symptoms, rescue medication use, frequency and severity of exacerbations and lung function. Based on these criteria, 6 children were classified with occasional asthma, 20 with frequent asthma and 19 with persistent asthma, all of them with moderate persistent asthma except from only one child that was classified with moderate-to-severe persistent asthma. In one child we could not determine the asthma severity. Out of the total 46 children, 13 were normal-weight, 8 overweight, and 25 obese according to the age and sex-specific thresholds proposed by Cole [21]. All children were born in due term (≥37 weeks of gestational age), normal delivery, except one child who was born by cesarean.

The children’s parents or guardians were informed about the study and the sampling procedure and written consent was obtained. The study protocol was approved by the local Ethics Committee of Granada (Reference 8/15) and was conducted according to the standards given in the Declaration of Helsinki (Edinburg 2000 revised), the Good Clinical Practice of the European Union (document 111/3976/88 July 1990) and legal in-forced Spanish regulations, which regulated the clinical investigation in human beings.

Clinical data and biological samples were collected by qualified personal and were codified according to the biobank of the Public Health System of Andalusia (BBSSPA) guidelines. Stool samples were collected by the children’s parents or guardians, placed into the provider plastic bottle and kept at −20 °C until and delivered to the hospital. All samples were kept at −80 °C in the BSSPA facilities until analysis. Blood samples were collected after 12 h of fasting by venipuncture in a standard hospital anticoagulant (EDTA) coated tubes. Plasma samples were prepared by centrifugation of blood samples at 1500× *g* for 10 min at 4 °C, then plasma samples were centrifuged at 2500× *g* for 15 min at 4 °C and kept in −80 °C at the BSSPA biobank facility. Plasma samples were used for the determination of blood inflammatory biomarkers and metabolomics analyses.

A total of 104 non-asthmatic children (30 normal-weight, 18 overweight, and 56 obese children) recruited in the Hospital Reina Sofia of Cordoba, described elsewhere [22] were used as a control group to determine the main basal biochemical and anthropometric differences between allergic asthmatic and non-asthmatic children.

### 2.2. Determination of Clinical, Biochemical and Anthropometric Parameters

Trained pediatricians recorded a complete allergic asthma clinical history by a standard semi-structured clinical interview. Allergic status was determined by a positive serum specific IgE test and by a standard skin prick test. Additionally, a spirometry analysis was performed according to the American Thoracic Society criteria [23]. Three forced vital capacity (FVC) maneuvers were performed and the best value of FVC and forced expiratory volume in 1 s (FEV1) and 25 s (FEV25) was recorded. The spirometry test was not considered an inclusion criterion because of in children FEV1 does not correlate well with the magnitude of asthma symptoms [24]. Similarly, exhaled nitric oxide fraction was not determined because it decreases after treatment with inhaled corticosteroids [25].

Bodyweight (kg), height (cm) and waist circumference were measured using standardized procedures. BMI was calculated as the weight divided by the square root of the height (m^2^). Systolic and diastolic blood pressures were measured three times by the same examiner using a mercury sphygmomanometer and following international recommendations [26].

Protein C reactive (PCR), transferrin, vitamin D, glutamate oxalacetate transaminase (GOT), glutamate pyruvate transaminase (GPT), gamma glutamyl transferase (γGGT), HDL cholesterol, LDL cholesterol, total cholesterol, triacylglycerol, glucose, insulin, sodium, potassium, calcium, uric acid, and thyroid-stimulating hormone levels were determined by routine biochemical analysis. Besides a hematologic analysis was also performed. Both analyses were performed in the Central Laboratory Analysis Unit for both hospitals following internationally and accepted quality controls. The Homeostatic Model Assessment for Insulin Resistance (HOMA-IR) index was calculated using fasting plasma glucose and insulin levels.

### 2.3. Inflammatory Biomarkers Determination

Plasma adipokines (adiponectin, leptin and resistin), as well as inflammatory biomarkers interleukin (IL) 4, 5, 6, 8, 10, 13, tumor necrosis factor-alpha (TNF-α), monocyte chemoattractant protein 1 (MCP-1) were analyzed on a Luminex 200 system (Luminex Corporation, Austin, TX, USA) with human monoclonal antibodies (EMD Millipore Corp, Billerica, MA, USA) using MILLIplexTM kits (HADK1MAG-61K, HSTCMAG-258K, and HADK2MAG-61K) according to the manufacturer’s recommendations.

LPS-binding protein (LBP) and chitinase 3-like 1 (CHI3L1) levels were determined in plasma samples using CSB-EO9629H (CUSABIO TECHNOLOGY LLC, Houston, TX, USA) and DC3L10 (R&D Systems, InC, Minneapolis, MN, USA) ELISA kits, respectively, following the manufacturer’s instructions.

The coefficients of variation (CV) were 4.9, 8.0, 7.1, 6.3, 6.9, 10.5, 19.8, 20.8, 8.9, 15.2, 6.8, 10.4 and 11.5 for adiponectin, resistin, leptin, MCP-1, TNF-α, IL-10, 13, 4, 5, 6, 8, chitinase 3-like 1 and LBP, respectively.

### 2.4. 1H NMR Spectroscopy of Blood Plasma

One-dimensional proton nuclear magnetic resonance (1D 1H NMR) spectra of blood plasma samples were measured as previously described [27]. Briefly, plasma samples were thawed at room temperature and then 350 μL of plasma was carefully mixed with an equal volume of phosphate buffer in a 2.0 mL cryovial. An amount of 600 μL of the mixture was then transferred into SampleJet tubes (Bruker BioSpin, Rheinstetten, Germany) of L = 103.5 mm and O.D. = 5.0 mm. 1D 1H-NMR spectra were measured using a Bruker AVANCE III 600 MHz NMR spectrometers (Bruker Biospin, Rheinstetten, Germany) at the Department of Food Science (University of Copenhagen). The spectrometer was equipped with an automated sample charger SampleJet (Bruker BioSpin) with sample cooling and preheating station, a 5 mm inverse probe with z-gradient, and automated tuning and matching and cooling unit BCU-05. The spectra were recorded using the standard pulse sequence with water suppression (noesygppr1d) from the Bruker pulse program library. After 4 dummy scans, 32 scans were acquired generating free induction decays (FID) of 64k data points using a spectral width of 20 ppm. The relaxation delay and mixing time were set to 2.73, 4.0 and 0.01 s, respectively. The receiver gain was set to 90.5 for all of the experiments. The automation program was used for acquisition routines including locking, automatic tuning and matching, and shimming, pulse calibration, and optimized presaturation power for each sample. FIDs were Fourier transformed after multiplied with an exponential function corresponding to a line broadening of 0.3 Hz, followed by automated phasing and baseline correction. Raw 1H NMR was then converted into a metabolite table using SigMa [28].

### 2.5. Gas Chromatography-Mass Spectrometry (GC-MS) Based SCFA Analysis

Short-chain fatty acids (acetic, propionic, isobutyric, butyric, isovaleric, 2-metilbutyric and valeric acid) were determined by Gas Chromatography-Mass Spectrometry as reported previously [29]. In brief, 600 µL of 0.3 M oxalic acid was added to 300 μL of plasma, vigorously vortexed for 1 min, and then centrifugated at 2800× *g* for 15 min at ambient temperature. Subsequently, 240 μL were filtered through 0.45 μm pore size Ultrafree-MC-HV filters (Millipore, Cork, Ireland), and 180 μL of the filtrate was transferred into HPLC vials containing 9 μL of the internal standard 50 mM 2-ethylbutyric acid in water. The samples were analyzed using Gas Chromatography-Mass Spectrometry (GC-MS) instrument consisting of Agilent 7890A GC and an Agilent 5973 series MSD (Agilent, Waldbronn, Germany). An amount of 1 µL aliquot was injected into a split/split-less inlet of GC-MS at 285 °C, at the split ratio of 2:1. Mass spectra of selected ions, 41, 43, 45, 57, 60, 73, 74, and 84 *m*/*z*, were recorded at a dwell time of 50 ms. The MS detector was switched off during the first one minute of the run. For detailed GC conditions see Weise et al. SCFA were identified in level 1, using authentic standards of SCFA purchased from (Sigma-Aldrich, Darmstadt, Germany). The final peak table of SCFA was generated by integrating the areas of corresponding GC-MS peaks in MATLAB (version R2015a, The MathWorks, Inc., Natick, MA, USA) and custom scripts written by authors. The peak table was normalized by the area of an internal standard prior to statistics.

### 2.6. Fecal D and L lactate and Intestinal Microbiota Analysis

Fecal D and L lactate were determined by colorimetric methods using the MAK058-1KT and MAK065-1KT, respectively, assays from Sigma-Aldrich (Merck Life Science, S.L.U, Damstadt, Germany).

We performed amplicon sequencing of 16S rRNA genes to analyze the composition of the intestinal microbiota. DNA was extracted from stool samples using a QIAamp DNA stool Mini Kit (QIAGEN, Barcelona, Spain) following the manufacturer’s recommendations, with the exception that samples were incubated with the lysis buffer at 95 °C instead of 70 °C to ensure the lysis of both Gram-positive and Gram-negative bacteria. The sequencing was performed according to the Illumina 16S Metagenomic Sequencing Library Preparation protocol at the facilities of the Department of Microbiology, University Hospital Campus de la Salud (Granada, Spain). The V3-V4 region of the bacterial 16s rRNA gene was amplified using the primers described by Klindworth et al., 2013 [30].

Bioinformatics analysis was performed with the “Quantitative Insights Into Microbial Ecology 2” (QUIIME2) pipeline, v. 2019.4 [31]. The data were denoised with the q2-dada2 plugin, which implements the DADA2 R library [32], performing sequence quality control, truncation of the reads, stitching R1 and R2 reads, generation of amplicon sequence variants (ASV) and screening out potentially chimeric sequences. Taxonomy was assigned using the QIIME 2 Naïve Bayes method with the GreenGenes (v. 13.8) database as a reference [33]. Alpha diversity (Shannon’s diversity index) and beta diversity (Unifrac index) analysis were performed using the q2-diversity plugin after samples were rarified (subsampling without replacement to 33,721 reads per sample). Kruskal–Wallis tests followed by Storey’s FDR correction were used to determine whether significant differences existed across sample groups at phyla level by using the software STAMP v.2 [34] For the detection of differences in microbial taxonomic composition at lower taxonomic levels we used the method “Analysis of Composition of Microbiomes” (ANCOM) [35], which considers the compositional nature of microbiome data, as implemented through the QIIME2 ANCOM plugin.

### 2.7. Statistical Analysis

Before the integrative analysis, a statistical analysis was performed to determine the characteristic of allergic asthmatic children, based on their obese status (normal-weight, overweight, and obese). For those variables following a normal distribution, an analysis of variance (ANOVA) test and Bonferroni post hoc test were performed. For those variables not following a normal distribution the one-way non-parametric ANOVA Kruskal–Wallis test was applied. The results in the tables are presented as the mean ± standard deviation (SD) unless otherwise indicated.

The baseline characteristics between children with asthma (BIOASMA cohort) and non-asthmatic children (Control cohort) were compared using a Student *t*-test for those variables following a normal distribution or a Mann–Whitney test for those variables not following normal distribution was performed. Statistical analyses were performed using SPSS version 19, for Windows (SPSS, Chicago, IL, USA).

### 2.8. The Multi-Omics Approach

Two specific analyses were performed to identify differences among the allergic asthmatic children cohort: (1) based on their obese status (normal-weight, overweight, and obese classification) and (2) based on their asthma severity.

**Figure 1:**
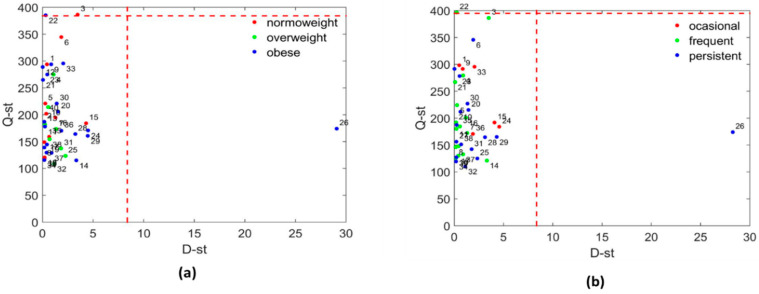
Multivariate Statistical Control Chart based on Principal Components Analysis. Hotelling T2 (D-st) versus the residual sum-of-squares (Q.st). Control limits are shown in red dashed lines. (**a**) Children were classified according to their obese status. (**b**) Children were classified according to asthma severity.

To perform a system biology approach we used the Multivariate Exploratory Data Analysis (MEDA) Toolbox in Matlab (https://github.com/josecamachop/MEDA-Toolbox). In a first approach, we combined the information of children’s characteristics (age, morphology, and allergies), clinical variables (biochemistry, fecal D and L lactate, hemogram, and inflammatory biomarkers), metabolomics (NMR, and GC-MS) and metagenomics data in a single matrix of data. This yielded a data matrix of 46 rows (children) and 224 columns (variables). Missing elements were imputed with unconditional mean replacement so that each missing value of a variable was replaced by the corresponding average computed from available readings. Outliers were identified by using Principal Component Analysis (PCA) [36,37], coupled with Multivariate Statistical Process Control (MSPC) [38] (Figure 1), and removed from all the subsequent analyses.

To identify the specific differences in the data, we applied Partial Least Squares-Discriminant Analysis (PLS-DA) [39] and its sparse variant (sPLS-DA) [40]. One discriminant model was fitted per each class versus the rest of the class, using both techniques. This resulted in three PLS-DA and three sPLS-DA models based on obesity classification and another three models for each technique based on asthma severity classification. Double cross-validation was applied to select the number of latent variables (LVs) in PLS-DA, and of non-zero loadings (NZLs, the number of selected variables) and LVs in sPLS-DA. We did not find any relevant correlation with the sex variable and, for this reason, we did not control for sex in any of the analyses.

## 3. Results

### 3.1. General Characteristics of the Participants

The clinical characteristics of the allergic asthmatic children (the BIOASMA cohort) are shown in Table 1, whereas in Table 2, we show the plasma inflammatory biomarker concentrations in the same cohort.

After that, we decide to characterize the asthmatic cohort with respect to non-asthmatic children, using a well-known cohort [22]. The clinical, biochemical, and inflammatory biomarkers in the BIOASMA cohort compared to the control population are summarized in Table 3. In general terms, the allergic asthmatic children present lower plasma values of leptin and IL-6. This may be since control children present a higher degree of obesity. Additionally, an increase in inflammation is observed in the asthmatic children (BIOASMA cohort), with higher plasma values of uric acid, leukocytes and MCP-1, and lower levels of adiponectin, than in the control cohort.

### 3.2. 1H-NMR Metabolites and Intestinal Microbiota Composition in the Asthmatic Cohort

Before the multi-omics analysis, ^1^H-NMR metabolites and the intestinal microbiota datasets were independently analyzed. ^1^H-NMR spectra obtained from the plasma samples correspond to 29 and 25 known and unknown metabolites, respectively (Table S1). A significant lower acetate relative proportion was found in obese asthmatic children compared to normal-weight asthmatic children (Kruskal–Wallis test, *p* = 0.038). In terms of asthma severity, higher creatinine relative proportions were found in the persistent group compared to the occasional and frequent group (Kruskal–Wallis test, *p* = 0.004).

We analyzed the intestinal microbial communities of the asthmatic cohort by barcoded sequencing (taxonomic composition is shown in Figure S1). In terms of alpha diversity (Shannon index), the analysis only found marginal significant differences between normal-weight and obese children (Kruskal–Wallis test, *p* = 0.09) as well as between obese and overweight (Kruskal–Wallis test, *p* = 0.07) children (Figure S2). No differences were found between subjects with different levels of asthma severity (Kruskal–Wallis test, *p* = 0.882). However, significant differences were found in beta diversity (unweighted UniFrac) between normal-weight and obese children (PERMANOVA test, *p* = 0.003) (Figure S2), suggesting differences in microbial community composition. We also did not find significant differences in beta diversity between children with different levels of asthma severity (PERMANOVA test, *p* = 0.47).

We, therefore, investigated differences in microbial taxonomic composition focusing the analysis to compare normal-weight and obese children. As expected, the most abundant phyla by far in the intestinal samples were Firmicutes and Bacteroidetes (Figure S1), with a higher relative abundance of Firmicutes (Kruskal–Wallis test, *p* = 0.041) and Actinobacteria (*p* = 0.037) in the obese children (Figure S1). We performed an Analysis of Composition Microbiomes (ANCOM) to identify lower-level taxa that differed in relative abundance between normal-weight and obese groups. ANCOM revealed several differential abundance taxa at the family level, highlighting the higher relative abundance of *Coriobacteriaceae*, *Streptococcaceae*, *Peptostreptococcaceae*, and *Erysipelotrichaceae* families in the obese children, whereas they presented a lower relative abundance of unclassified family members of the *Clostridiales* and RF32 order, belonging to the *Clostridia* and *Alphaproteobacteria* classes, respectively (Table S2).

### 3.3. Systems Biology Characterization of the Asthmatic Cohort

**Figure 2:**
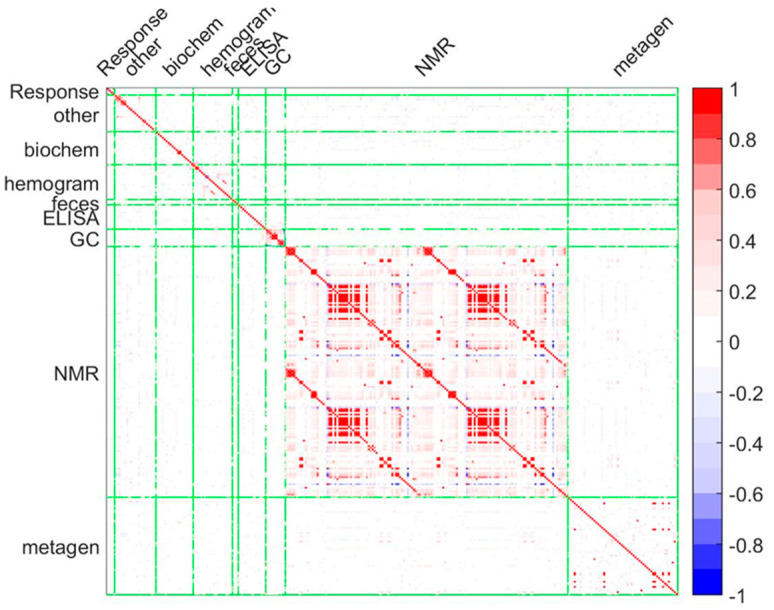
Multivariate Exploratory Data Analysis (MEDA) correlation map. MEDA technique of the predictive correlations was performed [41]. Green lines represent the divisions between the response and the different datasets. Biochem: Biochemical data; Hemogram: hemogram data; Feces: Lactic D and L acid; NMR: nuclear magnetic resonance data (noesy and cpmg data); GC: gas-chromatography short-chain fatty acids data; Metagen: metagenomics data; ELISA: Elisa and luminex data.

We found the highest correlations of variables within the metabolomics and metagenomics data blocks, and very little cross-correlation between data blocks (Figure 2). The correlation is obtained with the MEDA algorithm, which allows us to obtain predictive correlations. A predictive correlation shows to what extent the content of one variable can be predicted from another. This type of prediction is similar but safer that Pearson’s correlation because the MEDA correlation tends to shrink low correlations to 0.

In Figure 2, we can observe that the correlation between blocks, in the areas outside of the diagonal, are all almost completely white (absence of correlation), but there are significant correlations within blocks, e.g., in NMR and metagenomics. This shows that both sources of information provide complementary information to understand the differences among individuals, but also suggests that both sources of information may be relevant to understand the disease and that should be included in an integrated model.

#### 3.3.1. Systems Biology Characterization of the Asthmatic Cohort According to the Obese Status

**Figure 3:**
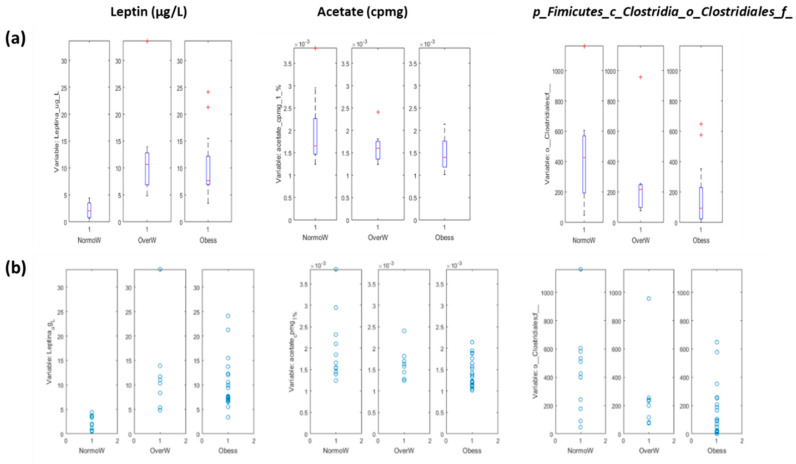
Representation of the variables associated with the obese status in allergic asthmatic children. (**a**) Box plot representation. Data are expressed as the mean and quartiles; (**b**) Dots plot representation. NormoW: normal-weight; OverW: overweight; Obess: Obese.

After applying the aforementioned discriminant models, when children were classified according to their obesity degree, we only found significant results (*p* < 0.05) for the sPLS-DA model between normal-weight and the rest (overweight and obese) (2 LVs and 4 selected variables, Area Under the Receiver Operating Characteristics (ROC) curve 0.75 ± 0.09). The sPLS-DA model identified leptin, acetate, and the unclassified bacteria family from the *Clostridiales* order as the variables associated with the difference (Figure 3). We did not find any association with SCFA measured by gas chromatography probably because the signal was very low in our samples.

#### 3.3.2. Systems Biology Characterization of the Asthmatic Cohort According to the Asthma Severity

**Figure 4:**
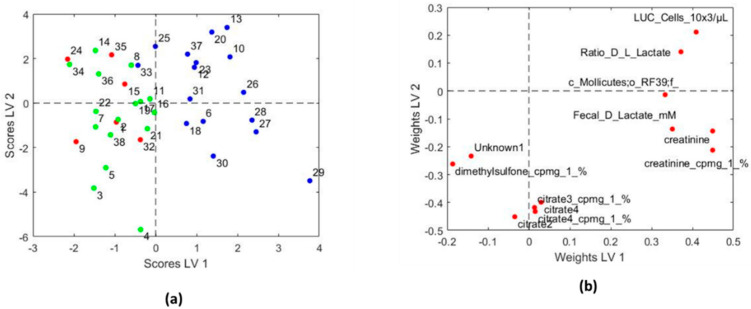
PLS-DA model from the variants selected by sPLS-DA model in terms of asthma severity. (**a**) score plot of the first two latent variables (LVs); Blue: Asthmatic children with persistent asthma, Green: Asthmatic children with frequent asthma, Red: Asthmatic children with occasional asthma; (**b**) weights plot of the first two LVs. Asthmatic children with persistent asthma showed higher values of the variables towards the upper right corner of the weights plot, and lower values of the rest, compared to the asthmatic children with occasional plus frequent asthma. Citrate 2–4 are several signals of the same metabolite. PLS-DA: Partial Least Squares Discriminant Analysis; sPLS-DA: sparse variant of the Partial Least Squares Discriminant Analysis; cpmg: metabolites found from the NMR spectra recorded using CPMG (Carr–Purcell–Meiboom–Gill) pulse sequence; LUC: large unstained cells.

**Figure 5:**
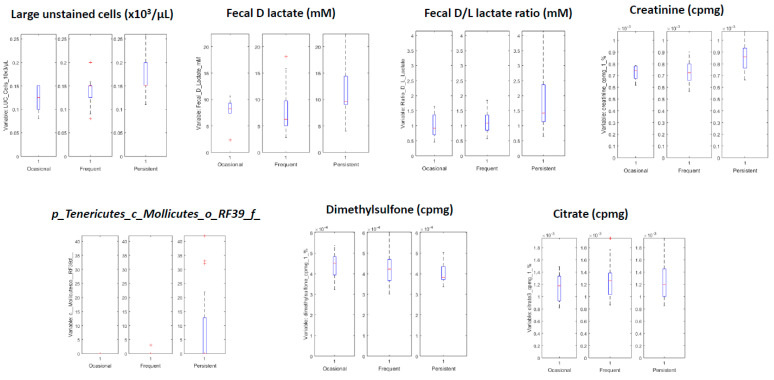
BOX plot representation of the variables associated with asthma severity (Occasional, frequent and, persistent). CPMG (Carr–Purcell–Meiboom–Gill) pulse sequence.

In the case of asthma severity, we only found significant results for the sPLS-DA model between children with persistent asthma and the rest (2 LVs and 13 selected variables, Area Under the ROC 0.66 ± 0.08). The resulting model was more complex than in the previous case, with a total of 12 variables selected (Table 4). We used PLS-DA to identify whether those variables presented statistically higher or values for children with persistent asthma, in comparison to the other children. The resulting model is shown in Figure 4. In Figure 5, we show the box plot for the variables. The association of creatinine, citrate, dimethylsulfone and c_*Molliutes_RF39;f* with asthma severity were not found in the previous analysis. We did not find a clear grouping of the individuals due to the obesity index in the model that predicts asthma severity.

## 4. Discussion

In the present study, we found that the obese allergic asthmatic phenotype in children was markedly associated with higher levels of leptin and lower relative proportions of plasma acetate and unclassified family from the *Clostridiales* order. The present study also found that allergic children with a worse asthma outcome presented higher levels of LUC cells, fecal D lactate and D/L lactate ratio, a higher relative proportion of plasma creatinine, and an unclassified family member from the RF39 order belonging to the *Mollicutes* class. Additionally, these children had lower plasma proportions of citrate and dimethylsulfone metabolites.

In the last years, the multi-omics approach has emerged as an important tool in the study of complex biological systems. In our case, we included data from the most basic clinics to subsequent inflammatory proteins, metabolites, and metagenomics data to decipher the implication of obesity in childhood allergic asthma. The obtained results indicate that there is no association between obesity and asthma severity in allergic children. Although our sample size is small, according to the data distribution, we can state that this does not affect the outcome. Similar results have been described by other authors, which show that increased childhood adiposity or obesity is only associated with non-Th2 asthma [42,43].

Nevertheless, our analysis shows that obese allergic asthmatic children present certain features that allow them to be distinguished from the normal-weight phenotype. That is, higher leptin levels and a lower relative proportion of acetate and an unclassified family of the *Clostridiales* order. Recently, in the KOALA population [44], an inverse association has been described between an uncultured *Clostridiales* II and BMI and weight z-score. Furthermore, previous studies in allergic adults, to nuts and seasonal pollen, described a reduced *Clostridiales* and increased *Bacteroidales* in their intestinal microbiota [45]. The SCFA acetate is produced in the colon by the intestinal bacteria, although other endogenous plasma sources include fatty acid oxidation, amino acid metabolism, or ketogenesis [46]. Acetate has been demonstrated to protect from allergic airway inflammation in animal models [15]. Therefore, it seems that obesity is associated with parameters related to increased allergic airway inflammation.

As a secondary outcome, we wanted to characterize allergic asthma phenotype by means of asthma severity. This analysis reveals that children with persistent asthma have an altered inflammatory profile, energy metabolism, and intestinal microbiota composition. Persistent asthmatic children have a higher number of LUC cells which are peroxidase-negative cells that do not fit into other categories of leukocytes. These types of cells normally include virally activated lymphocytes, plasma cells, hairy cells, pediatric lymphocytes, and peroxidase-negative blast [47]. In recent years, it has been suggested that an increase in the percentage of LUC cells may reflect heightened immune activation in HIV infection [47]. In our study, we found an increased relative proportion of plasma creatinine and lower citrate metabolites in persistent asthmatic children. It has been previously described that creatinine is involved in muscular protein turnover and energy supply to muscles, such as the airway smooth muscles [48]. On the other hand, different studies have described that the tricarboxylic cycle pathway is associated with asthma [49,50].

Numerous studies have pointed out that alterations of the intestinal microbiota and its metabolites are linked to changes in the immune response and inflammation together with disease development in the lungs [51]. Children with persistent allergic asthma present a higher relative proportion of an unclassified family from the *Mollicutes* class. They also have higher fecal D lactate concentration and D/L lactate ratio and lower proportion of the plasma dimethylsulfone metabolite. Lactate is a key intermediate for the gut fermentation. The ratio D/L lactate has been implicated as a marker for microbiota imbalance in patients with short bowel syndrome, an inflammatory condition of the gastrointestinal tract [52]. Finally, dimethylsulfone (DMSO2), is a common metabolite that can originate from different sources, including dietary supplementation. DMSO2 have an endogenous origin through the methionine metabolism, but can also be produced by the decomposition of methionine by the intestinal microbiota [53]. Taken together, these results could indicate an altered gastrointestinal microbiota in children with persistent allergic asthma.

A major limitation of our study is the low number of children included because of the intestinal microbiome and the metabolomics profile can be affected by different factors. In an attempt to minimize this risk, we established a stringent set of exclusion criteria (use of medication, preterm children, diagnosis of other diseases). Moreover, the asthmatic cohort is homogenous in terms of geographical area, population (all are children) and asthma diagnosis. Moreover, our results are based on the sPLS technique that enables the selection of the most predictive or discriminant variables in high throughput data that are characterized by thousands of variables and a small number of samples [40].

## 5. Conclusions

Overall, our integrative analysis identified a specific association between metagenomics, metabolomics and hematological parameters in pediatric allergic asthma. Our findings confirm the molecular heterogeneity of the allergic asthma phenotype, while also highlighting the utility of using high-throughput technologies and the multi-omics approach to examine allergic asthma at a more holistic level. Future works are needed to validate the implication of the described taxon and metabolites in the pediatric allergic asthma outcome and treatment.

## Figures and Tables

**Table 1:** Anthropometric, clinical, biochemical and hematologic characteristics of the BIOASMA cohort.

	Normal-Weight	Overweight	Obese	*p* Value
N(F/M)	13 (3/10)	8 (2/6)	25 (7/18)	-
Age (year)	8.5 ± 3.0	9.6 ± 2.0	8.4 ± 2.0	0.4
**Anthropometric characteristics**				
BMI	16.1 ± 1.3	22.6 ± 1.9	24.3 ± 2.6	0.001 ^a,b,c^
Hip circumference (cm)	69.4 ± 7.3	83.7 ± 7.9	82.4 ± 10.2	0.003 ^a,b^
BMIzscore	−0.5 ± 0.7	1.7 ± 0.2	2.7 ± 0.7	0.001 ^a,b,c^
Waist to hip ratio	0.8 ± 0.0	0.8 ± 0.1	0.9 ± 0.1	0.3
Systolic blood pressure (mmHg)	100 ± 11.5	108.7 ± 15	109.2 ± 12.9	0.2
Diastolic blood pressure (mmHg)	56.3 ± 5.3	59.2 ± 9.7	64.6 ± 12.5	0.1
**Asthmatic related clinical parameters**				
GEMA classification (occasional/frequent/persistent)	2/7/3	0/2/6	5/10/10	-
IgE higher than 3.5	13	7	21	-
FEV1 (%)	93.3 ± 14.3	100.9 ± 8.6	99.8 ± 12.7	0.7
FVC (%)	96.1 ± 13.0	96.3 ± 6.3	99.0 ± 11.7	0.7
FEF25-75 (%)	80.9 ± 23.0	91.6 ± 22.3	86.4 ± 23.3	0.7
FEV1/FVC ratio	93.5 ± 14.0	97.4 ± 12.0	92.2 ± 12.1	0.7
**Biochemical and hematological parameters**				
Glucose (mg/dL)	84.2 ± 9.7	88.6 ± 7.1	85.2 ± 9.5	0.05
Insulin (μU/mL)	6.4 ± 3.7	11.3 ± 2.5	12.7 ± 18.4	0.028 ^b^
HOMA index	1.1 ± 0.6	2.25 ± 0.3	2.9 ± 5.5	0.011 ^b^
Triacylglycerols (mg/dL)	53.7 ± 13.7	78.7 ± 31.0	76.5 ± 36	0.1
HDL (mg/dL)	55.6 ± 10.4	56.7 ± 14.2	52.2 ± 10.0	0.6
LDL (mg/dL)	80.6 ± 18.3	99.2 ± 10.0	96.5 ± 23.5	0.2
Total cholesterol (mg/dL)	148.4 ± 20.0	164.7 ± 23.4	159.8 ± 29	0.4
C reactive protein (μU/mL)	5.0 ± 7.5	2.4 ± 2.3	4.9 ± 4.9	0.6
Transferrin (mg/dL)	302.1 ± 64.3	287.6 ± 26.5	287.8 ± 32.2	1.0
GPT	16.5 ± 4.4	19.6 ± 8.1	25.1 ± 11.7	0.038 ^a^
γ-GT	14.6 ± 2.7	17.7 ± 3.3	17.1 ± 4.4	0.1
TSH (μU/mL)	2.3 ± 0.8	2.4 ± 0.8	2.4 ± 1.2	1.0
Uric acid (mg/dL)	4.1 ± 0.7	3.7 ± 0.8	4.7 ± 1.0	0.018 ^c^
Sodium (mEq/L)	136.8 ± 1.8	138.6 ± 1.4	138.2 ± 2.0	0.033 ^a^
Potassium (mEq/L)	4.5 ± 0.4	4.5 ± 0.5	4.4 ± 0.2	0.9
Calcium (mg/dL)	10.0 ± 0.2	10.2 ± 0.3	10.1 ± 0.3	0.1
Vitamin D (ng/mL)	24.9 ± 10.7	27.4 ± 8.2	26.1 ± 6.3	0.8
Erythrocyte (×10^6^/μL)	5.1 ± 0.4	4.9 ± 0.3	5.0 ± 0.2	0.3
Hemoglobin (g/dL)	13.5 ± 1.0	13.7 ± 0.8	13.7 ± 0.9	0.8
Leukocyte (×10^3^/μL)	7.9 ± 3.2	7.2 ± 2.2	7.9 ± 1.7	0.5
Neutrophils (×10^3^/μL)	3.9 ± 1.8	3.4 ± 1.3	3.7 ± 1.2	0.7
Linfocyte (×10^3^/μL)	2.6 ± 0.9	2.7 ± 0.6	3.0 ± 0.8	0.2
Monocyte (×10^3^/μL)	0.5 ± 0.2	0.5 ± 0.1	0.5 ± 0.2	0.9
Eosinophils (×10^3^/μL)	0.7 ± 0.8	0.5 ± 0.6	0.5 ± 0.5	0.5
LUC cells (×10^3^/μL)	0.2 ± 0.1	0.2 ± 0.1	0.1 ± 0.1	0.7
Platelet (×10^3^/μL)	306.9 ± 57.9	291.6 ± 90.7	320.2 ± 66.8	0.6

F, female; M, male; BMI, Body mass index; BMIzscore: Body mass index z score; IgE, immunoglobulin E; FEV1, forced expiratory volume in 1 s; FVC, forced vital capacity; FEF25-75, forced mid-expiratory flow rate; GPT, Glutamate Pyruvate Transaminase; γ-GT, γ-glutamyl transferase; HDL, High-Density Lipoprotein; LDL, Low-Density Lipoprotein; TSH, thyroid-stimulating hormone; HOMA index, Homeostatic Model Assessment for Insulin; LUC cells, Large Unstained Cells. ^a^ Differences between the normal-weight and obese groups, ^b^ differences between the normal-weight and overweight groups and ^c^ differences between the overweight and obese groups. Significant differences (BMI, hip circumference and BMIz-score) were determined by an analysis of variance (ANOVA) test or a Kruskal–Wallis test (Insulin, HOMA index, GPT, uric acid and sodium).

**Table 2:** Adipokines and inflammatory biomarkers in the BIOASMA cohort.

	Normal-Weight	Overweight	Obese	*p* Value
Adiponectin (mg/L)	7.0 ± 4.3	6.9 ± 3.8	6.8 ± 4.4	0.9
Resistin (μg/mL)	13.8 ± 4.3	13.5 ± 2.8	13.4 ± 4.8	1.0
Leptin (μg/L)	2.2 ± 1.4	12.4 ± 9.1	10.0 ± 4.8	0.0001 ^a,b^
MCP-1 (pg/mL)	124.0 ± 35.8	115.6 ± 28.9	134.3 ± 54.2	0.6
TNF-α (pg/mL)	4.0 ± 1.1	3.7 ± 2.0	3.5 ± 1.0	0.3
Chitinase-3 (μg/L)	17.1 ± 7.6	19.4 ± 6.2	21.5 ± 8.8	0.4
IL-10 (pg/mL)	4.0 ± 1.5	5.7 ± 2.4	4.5 ± 1.7	0.3
IL-13 (pg/mL)	3.0 ± 1.8	3.1 ± 2.7	2.3 ± 2.6	0.4
IL-4 (pg/mL)	13.5 ± 6.3	39.9 ± 52.8	13.4 ± 6.7	0.2
IL-5 (pg/mL)	1.3 ± 0.4	1.7 ± 1.0	1.4 ± 0.4	0.7
IL-6 (pg/mL)	1.6 ± 1.3	1.7 ± 1.3	1.7 ± 2.3	0.8
IL-8 (pg/mL)	2.1 ± 0.6	1.7 ± 0.5	1.8 ± 0.7	0.5
LBP (µg/mL)	3.0 ± 1.4	3.8 ± 1.2	3.5 ± 1.8	0.6

MCP-1, monocyte chemoattractant protein 1; TNF-α: Tumor necrosis factor-alpha; IL: Interleukin; LBP: lipopolysaccharide-binding protein. ^a^ Differences between the normal-weight and obese groups, ^b^ Differences between the normal-weight and overweight groups with the non-parametric Kruskal–Wallis test.

**Table 3:** Characteristics of the BIOASMA cohort compared to control children.

	Asthmatic Children	Control Children	*p*-Value
N(F/M)	46 (12/34)	104 (57/47)	-
Age (year)	8.7 ± 2.3	8.0 ± 0.5	0.1
BMI	21.6 ± 4.2	22.5 ± 5.4	0.4
BMIzscore	1.6 ± 1.5	2.1 ± 2.0	0.2
Systolic blood pressure (mmHg)	107.3 ± 13.2	107 ± 12.5	0.9
Diastolic blood pressure (mmHg)	62.1 ± 11.3	67.9 ± 12.9	0.005
GPT (U/L)	22.0 ± 10.4	20.5 ± 8.3	0.8
γ-GT (U/L)	16.6 ± 4.0	14.9 ± 9.6	0.001
Triacylglycerols (mg/dL)	71.3 ± 32.2	64.6 ± 26.6	0.3
HDL (mg/dL)	53.8 ± 10.7	57.6 ± 15.6	0.3
LDL (mg/dL)	93.2 ± 21.3	94.6 ± 25.2	0.8
Total cholesterol (mg/dL)	157.9 ± 26.4	165.1 ± 29.5	0.2
TSH (μU/mL)	2.4 ± 1.0	2.3 ± 1.0	0.4
Glucose (mg/dL)	85.5 ± 9.1	82.5 ± 7.4	0.1
Insulin (μU/mL)	10.9 ± 14.4	7.9 ± 4.6	0.1
HOMA index	2.3 ± 4.3	1.6 ± 1.0	0.1
Uric acid (mg/dL)	4.4 ± 1.0	4.0 ± 1.0	0.02
Hemoglobin (g/dL)	13.7 ± 0.9	13.2 ± 3.1	0.000
Leukocyte (×10^3^/μL)	7.8 ± 2.2	7.0 ± 2.0	0.02
Adiponectin (mg/L)	6.9 ± 4.2	22.7 ± 11.0	0.000
Resistin (mg/L)	13.5 ± 4.3	13.2 ± 8.7	0.05
Leptin (µg/L)	8.5 ± 6.5	16.4 ± 14.10	0.005
MCP-1 (pg/mL)	128.2 ± 45.9	103.9 ± 61.2	0.001
TNF-α (pg/mL)	3.7 ± 1.2	3.9 ± 2.3	0.9
IL-6 (pg/mL)	1.7 ± 1.8	7.5 ± 12.5	0.002
IL-8 (pg/mL)	1.8 ± 0.6	2.4 ± 2.6	0.1

F, female; M, male; BMI, Body mass index; BMIzscore: Body mass index z score; GPT, Glutamate Pyruvate Transaminase; γ-GT, γ-glutamyl transferase; HDL, High-Density Lipoprotein; LDL, Low-Density Lipoprotein; TSH, thyroid-stimulating hormone; HOMA index, Homeostatic Model Assessment for Insulin; MCP-1, monocyte chemoattractant protein 1; TNF-α: Tumor necrosis factor-alpha; IL: Interleukin; Differences between the asthmatic and control children were determined by a Student *t*-test (uric acid) or a Mann–Whitney test (Diastolic blood pressure, γ-GT, hemoglobin, leukocyte, adiponectin, leptin, MCP-1, and IL-6).

**Table 4:** Variables selected by the sPLS-DA model in terms of asthma severity.

Variables with Higher Values for Children with Persistent Asthma	Variables with Lower Values for Children with Persistent Asthma
LUC cells (×10^3^/µL)	Citrate 3 (cpmg)
Fecal D lactate mM	Citrate 4 (cpmg)
Ratio D/L lactate	Dimethylsulfone (cpmg)
Creatinine (cpmg)	Citrate 2
Creatinine	Citrate 4
c_Mollicutes;o_RF39;f_	Unknown NMR metabolite

sPLS-DA: sparse variant of the Partial Least Squares-Discriminant Analysis; LUC: Large unstained cells; NMR: nuclear magnetic resonance; cpmg: metabolites found from the NMR spectra recorded using CPMG (Carr–Purcell–Meiboom–Gill) pulse sequence. Citrate 2–4 are several signals of the same metabolite.

## Supplementary Materials

The following are available online at https://www.mdpi.com/2227-9059/8/9/359/s1, Figure S1: Composition of the intestinal microbiota communities in the asthmatics cohort. NW: normal-weight; OB: obese; Panel A shows the taxonomic composition of the intestinal communities at the phylum level. Each column represents a participant. Panel B shows the differential abundance analysis of the microbial taxonomic composition at the phylum level. Figure S2: Alpha and beta diversity of the intestinal microbiota communities of the asthmatic cohort. NW: normal-weight; OB: obese; Panel A shows the alpha diversity and panel B shows the beta-diversity: Principal coordinates analysis (PCoA) Unweigthted Unifrac analysis Table S1: ^1^H-NMR metabolites (cpmg-[Carr-Purcell-Meiboom-Gill] absolute data; Table S2: Analysis of composition of microbiomes (ANCOM) abundance table. The table shows the relative proportions quartiles from the obese and normal-weight group.
