# Supplementary material for: A Multi-Omics Approach Reveals New Signatures in Obese Allergic Asthmatic Children
Source: Biomedicines. 2020 Sep 18;8(9):359. doi: 10.3390/biomedicines8090359 (PMC7555790; doi:10.3390/biomedicines8090359)
Supplement: Supplementary file 1 [file biomedicines-08-00359-s001.pdf]

Figure S1. Composition of the intestinal microbiota communities in the BIOASMA cohort. NW: normal-weight; OB: obese

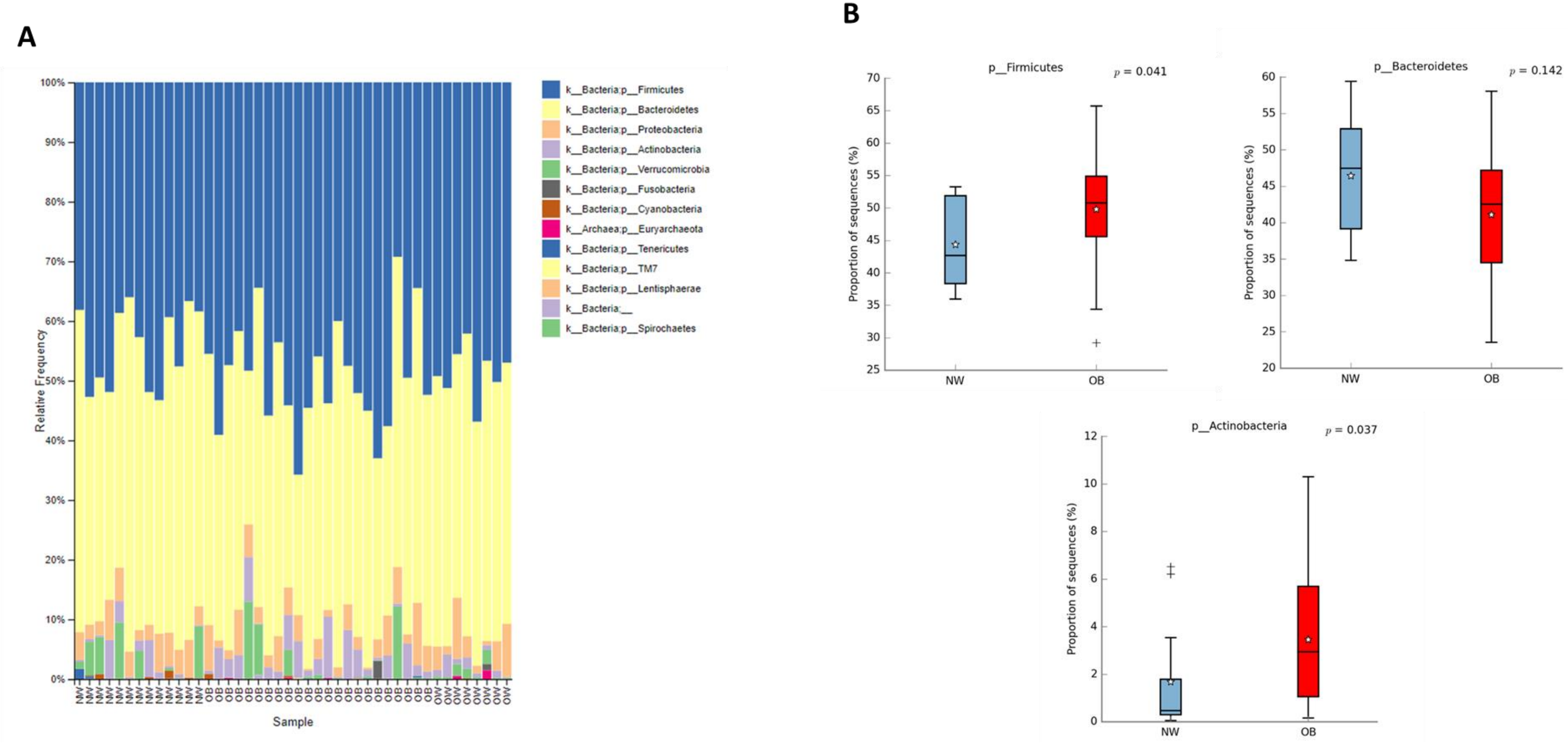

Figure S2. Alpha and beta diversity of the intestinal microbiota communities of the BIOASMA cohort NW: Normal-weight; OB: obese.

A. Alpha diversity measures by the Shannon Index

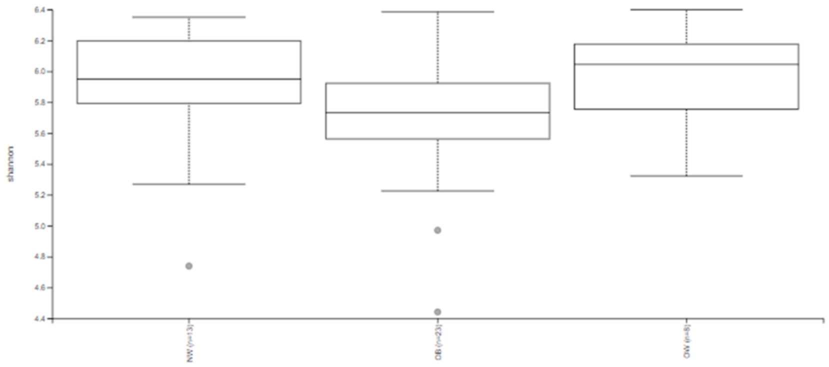

Kruskal-Wallis (pairwise)

Group 1	Group 2	H	p-value	q-value
NW (n=13)	OB (n=23)	2.876887	0.089860	0.134789
	OW (n=8)	0.047203	0.828004	0.828004
OB (n=23)	OW (n=8)	3.260870	0.070951	0.134789

B. Beta diversity measures by the unweighted Unifrac distances

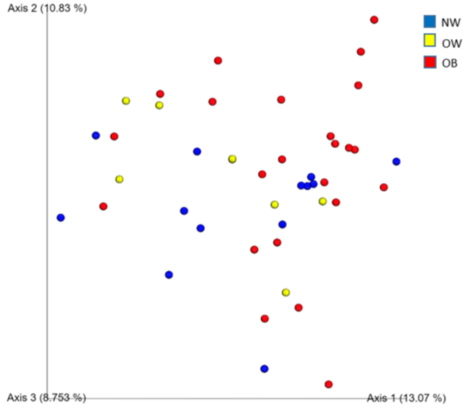

Pairwise PERMANOVA results

Group 1	Group 2	Sample size	Permutations	pseudo-F	p-value	q-value
NW	OB	36	999	2.077659	0.003	0.009
	OW	21	999	0.897867	0.597	0.597
OB	OW	31	999	1.290963	0.160	0.240

Table S1. H-NMR metabolites

Obese status	Normal-weight	Obese	Normal-weight	Overweight	Normal-weight	Normal-weight	Overweight	Obese	Obese	Overweight	Obese	Obese	Overweight	Obese	Normal-weight	Normal-weight	Obese	Normal-weight	Obese
Asthma status	Occasional	Frequent	Frequent	Frequent	Frequent	Persistent	Frequent	Frequent	Occasional	Persistent	Frequent	Persistent	Persistent	Frequent	Occasional	Frequent	Frequent	Persistent	Frequent
Metabolite																			
isoleucine1	88447	62237	83444	100853	64179	93635	75822	101039	58666	67291	62870	65076	73970	67827	53990	59278	66236	57017	79820
leucine	455640	360067	388930	487709	352720	522179	411875	445855	360671	357822	365224	361699	401498	355948	358179	394060	345983	335641	442994
valine1	655982	482759	583423	627840	470261	649663	640419	726448	528551	501086	557487	488425	579328	526878	504540	543125	471912	406100	666116
isoleucine2	126998	109321	106110	127441	88531	140484	107741	132062	105768	88001	100569	82744	113270	96780	99877	94827	101132	89766	134481
valine2	639546	472079	575706	624801	455395	629267	636805	701359	518384	488561	545802	477111	571328	496515	496182	522606	478084	403067	654402
isobutyrate	41862	22946	52305	54351	33155	55693	53191	56645	35062	64830	46230	31455	36952	31524	39204	41467	34403	35834	30723
ethanol	14136	16211	26075	19794	76605	22473	1286036	19874	204866	23913	16549	159302	20405	49629	14020	176574	15702	43021	17896
ethanol_3hydroxybutyrate	416164	54557	1048110	740944	298159	400974	593344	50197	120052	73272	54128	511124	59336	48003	107377	666352	49975	75672	48266
e	408977	47628	1043227	736654	267943	397113	24334	32507	24343	57513	46352	445035	48319	23711	103900	611373	39848	52076	43217
lactate1	3048430	5401394	4340500	4498960	2816501	6811795	5823144	5140813	6786438	5396901	4534964	3147956	4920687	4713585	7081522	8684252	3637236	4584886	5162429
alanine	481090	750865	337161	426006	438366	561466	740150	679294	879411	529887	541258	332299	532223	837033	530314	531143	460807	801411	767818
acetate	354320	99305	232161	223587	200040	123364	113971	140453	169525	162658	98482	137341	166774	136186	190668	142760	213820	178502	176030
succinate	129743	223927	52569	24911	137280	133434	218720	215717	213363	91848	62156	104297	141705	228628	263259	261952	157280	223464	237085
citrate1	112878	98937	132056	176591	129455	112171	112673	75796	116545	97575	105914	98817	73268	114268	121478	158218	106133	161721	104136
citrate2	100227	106236	133422	198703	135236	120905	114450	81885	108893	82874	83251	74283	61897	105184	135612	164160	138506	163554	86668
citrate3	116434	108214	138980	181569	138039	120314	120783	88278	120199	105472	107414	100909	80389	115547	135521	162744	105204	161325	104292
citrate4	181300	184926	211674	282638	209466	185024	196206	142229	189903	163823	164296	145179	135438	182348	212169	240903	177811	252023	166297
DMA	1923576	1882418	1745983	1883090	1755425	2024036	1716995	1862861	1763354	2001431	1803145	1830337	1771955	1746879	1794406	1968274	1771760	1995981	1928673
sarcosine	59529	30814	5200	31090	31447	71199	13732	6684	5983	2070	9454	26717	7569	7591	27163	18351	3562	8149	20111
DMG	51921	31001	28976	26208	40517	40745	31725	45989	28143	28058	24356	22663	16400	30339	34571	39327	27901	31968	31534
creatine_creatinine	39343	40927	51939	23877	34033	62237	39268	58158	46303	44573	38679	16964	24119	29806	81633	72085	34368	37925	37579
dimethylsulfone	49371	43643	47509	45222	46762	38362	41200	43068	43322	44723	30249	33982	34887	39539	48986	38864	40291	36779	40651
carnitine	16147168	15293400	12979345	15741413	16790346	13455984	14567369	18554526	14571929	19811234	15237820	15720324	16313325	6	25654476	21030560	19118032	19804547	16736485
TMAO	1144217	1058596	901257	1087202	1209395	1015011	1154028	1192319	1059670	1083838	965340	980049	1096094	1082727	963280	1051989	1080406	1234240	1131665
glucose1	1360880	1230836	1073958	1276540	1569863	1127462	1462217	1460533	1263349	1405373	1227300	1211980	1385615	1316089	1233491	1222386	1444968	1550016	1381185
methanol	47954	78554	64215	89504	77280	66293	71787	108198	107501	74540	87900	64826	66537	88742	88619	101120	48039	85742	71069
glucose2	2171384	1955257	1705375	1996171	2432954	1765596	2267429	2295377	1976786	2166251	1897489	1875058	2145536	2120241	1906391	1932959	2266530	2425509	2186341
acetoacetate2	21215	13288	19585	26446	13697	21643	13959	28487	22437	12933	29286	18933	22201	20368	19612	27877	12870	30316	19053
glucose3	990192	872475	749303	885420	1093102	809904	1010680	1013730	860275	969257	819915	812276	927661	924398	862477	854413	1010335	1099687	956111
glucose4	1391506	1246006	1103074	1281839	1560579	1136669	1455850	1470489	1267236	1393237	1214346	1215595	1372595	1352778	1225477	1237805	1452507	1556730	1402851
glucose5	1802400	1617543	1416334	1622954	1997095	1436612	1875470	1904116	1613262	1791993	1559796	1605676	1732688	1798640	1669214	1643751	1889783	1979879	1847202
glucose6	696869	620708	549122	639620	779278	560019	722751	725016	634621	690986	595553	594706	678213	674831	608161	616351	721626	781091	698484
glucose7	1066927	943566	862759	1008355	1198201	874842	1102011	1136955	963570	1062064	915706	896883	1038847	1078535	952056	977862	1112857	1209661	1110849
glycine	427717	616766	352506	370703	513960	521298	468641	375972	481526	444935	369219	285756	405525	416391	511075	459790	511931	563252	426084
glucose8	2205934	1982660	1763405	2073083	2471471	1812563	2292568	2338473	2018190	2221514	1938147	1913403	2226935	2202899	1941069	1988552	2240205	2469044	2194993
glucose9	390913	339755	233268	264670	323735	189647	414059	373786	201938	268035	208651	317580	268824	306739	436048	323270	572705	289478	585911
glucose10	691903	631277	565517	646813	765845	559644	731004	746097	646439	691512	611445	597197	690870	723071	602389	620946	842184	789083	788587

glucose11	1432601	1283303	1138155	1292740	1582664	1127784	1475725	1485395	1307911	1393359	1247435	1260733	1397302	1390583	1240634	1290389	1464998	1567467	1447318
glucose12	1823891	1648992	1462198	1690963	2039889	1509189	1903203	1949141	1686634	1811415	1638583	1636383	1816051	1803202	1599793	1642786	1896547	2023363	1858220
creatine	168958	124823	144766	120659	135049	142219	105151	132959	106457	106668	150037	91407	141244	90560	97748	96412	89409	115969	163572
creatinine	72455	64073	58636	83568	79537	73454	58653	77927	66252	94257	62620	79847	71353	68589	96990	81629	86331	88350	66726
lactate2	638043	1142291	901759	954435	602009	1421563	1241566	1097365	1466936	1153544	959973	667179	1050407	1000706	1486110	1837243	753025	970927	1077884
glucose13	1421268	1285177	1162007	1320802	1599228	1145161	1492272	1523731	1315676	1424363	1269706	1278501	1443184	1440741	1247601	1283917	1494326	1592351	1481720
urea	32937	25218	33220	30708	23534	25472	40383	36072	32757	35920	36593	26813	32493	22624	25929	29852	36861	24409	38994
tyrosine1	46369	76198	51773	57317	78174	75231	83369	107896	93448	83739	76255	44556	59362	79346	61131	58561	55165	66625	66063
1	61703	59665	53242	57688	59742	55707	69602	56590	69214	50610	44026	38567	65768	42047	60319	55416	47873	81648	55625
tyrosine2	50102	65341	46792	51981	71003	70869	76829	101085	86640	72891	68194	41053	51538	72529	57443	52118	54065	60889	60936
2	65806	63195	48566	60637	62661	50985	84528	61515	69444	48631	43880	38018	61707	46754	61801	62110	51371	79289	73463
formate	76356	69769	64864	78340	74932	67971	67786	81310	69956	57061	43275	47879	56200	60031	76675	70398	76583	68999	47008
unk1_s	17148	14579	24648	21376	20683	19836	22903	29699	17223	23982	27443	15755	17710	16435	23139	19533	21801	22908	23248
unk2_q	43867	103825	40346	42101	43015	48516	48460	43274	51584	43090	38827	42202	42846	38015	41816	41619	41533	36370	42117
unk3_d	80184	14839	190999	132265	47624	74811	10110	11603	6968	17475	11646	83639	11538	8574	23344	108423	14836	9625	17817
unk4_d	128385	17375	319264	230262	81417	126358	11780	13102	13238	24647	19178	139835	13451	12146	29892	185585	17631	19737	23707
unk5_d	25815	39838	25873	31680	30556	28935	40597	48788	51890	27759	45117	21771	31243	33285	27566	40030	28995	30953	35378
unk6_d	20822	33660	22167	26385	24271	23461	35216	46393	38037	28259	48105	26446	28569	35682	30345	31876	26971	24355	32086
unk7_d	117975	19711	303544	217603	79787	116685	12103	14416	13932	25280	20155	130537	19217	13412	28057	173953	16146	14421	15977
unk8_d	72922	20922	169498	119269	50694	70227	19917	18596	20495	23476	16403	76789	18013	18179	30743	106246	20425	22483	19932
unk9_s	4836443	4856460	4809709	5103227	4806657	4991056	4966447	4862082	4818859	4971550	4743410	4553374	4890023	4727825	4774883	4933215	4672930	4828091	4624801
unk10_s	9469	11767	6131	13231	7106	11540	10901	6065	8217	7719	13223	6852	11294	7132	8387	8605	4865	5598	8808
unk11_s	25274	28873	19045	28650	25881	28422	21424	15612	25427	22219	25003	24559	22293	23097	37079	19237	23712	26682	26026
unk12_s	446824	446121	440550	479819	448705	473903	458989	443656	451897	473800	452143	441846	477091	456288	452073	467727	427076	458764	430340
unk13_d	3855520	3883724	3858110	4037888	3850183	3987078	3985518	3906257	3848779	3992627	3837652	3743545	3927012	3780332	3803251	3916883	3790145	3842211	3751141
unk14_s	1371526	1056812	913882	1086389	1339055	1053273	1209473	1233070	1110850	1171550	1032395	1004596	1019386	1110217	1019300	1084730	1176756	1318788	1164865
e	54137	41281	62372	84824	43895	64559	57556	93124	80193	37628	103864	68174	66989	64448	63688	74430	56274	90677	74494
unk16_s	48786	43819	40942	96460	34886	56478	69652	81060	72039	39588	67422	61135	67261	79660	71171	189665	30735	85124	48781
unk17_s	176089	221888	143121	248142	176572	170251	237946	171214	349937	134886	154426	177245	134020	129870	163893	249577	142702	170826	177696
unk18_s	34612399	33273233	26171535	34233287	36147221	29131487	30650166	39297871	32005078	41103992	31644335	34418010	34419766	6	55854500	46563210	40036833	43380104	33692966
unk19_s	39529	36522	43832	60624	60890	51729	41045	54466	43442	60706	54649	39552	61375	68792	30697	41744	118758	71932	68789
unk20_s	105153	150842	90238	96115	110931	132277	160149	140880	162882	127427	116476	84218	109425	173752	129558	120955	117288	162725	155010
unk21_s	88197	74583	103745	112823	79786	106971	95070	140678	120509	77647	148094	110681	110469	113115	87293	116725	76498	120443	124563
unk22_d	68572	50973	53263	66537	57931	59180	66151	77311	67622	37429	72993	59890	45270	57515	65837	78745	54807	74060	74524
unk23_d	41507	39354	30535	41689	38487	51531	38403	49812	39319	37619	36097	38244	38060	35786	41386	35583	39465	42944	43902
unk24_d	38806	43358	32298	39729	39485	47497	36573	52874	38061	43324	37523	39822	36970	40710	42871	43059	42975	46061	44016
unk25_s	65	77	679	01	07	79	42	33	66	83	5	25	53	1023	94	54	706	32	18

Obese Persistent	Obese Frequent	Obese Persistent	Obese Occasional	Overweight Persistent	Obese Persistent	Normal-weight Persistent	Obese Persistent	Obese Persistent	Obese Persistent	Normal-weight Frequent	Obese Occasional	Obese Frequent	Overweight Persistent	Normal-weight Frequent	Normal-weight	Obese Persistent	Obese Frequent	Overweight Persistent
80328	71641	102941	90698	69585	83681	56681	77796	60592	91711	81265	66996	62881	78616	57403	59549	77133	68154	65704
355035	419018	548628	472464	407897	429755	335424	379524	370859	503488	312869	332531	409857	419320	331640	334378	442949	302289	388214
484291	557842	753852	608306	617197	629131	473501	626014	622476	744932	510207	483390	583592	576756	475812	516861	628322	478333	582586
91594	110830	176767	133690	118661	117674	95711	108943	91912	133146	83032	96196	105161	100138	86191	100907	127433	84500	109463
490999	559805	745205	625840	626089	608324	458127	613418	589640	734703	468627	483629	581327	555582	474712	532168	617930	459384	554788
69123	39081	43652	38589	33387	48476	27526	32961	38110	54123	47130	30657	37705	40156	39140	43152	53929	40222	45358
193242	22737	12960	176228	196219	19204	121909	736163	18639	25249	204814	195889	135200	43398	40960	142433	172842	20349	83796
319610	116237	57938	366870	104906	299038	171783	404287	69074	497637	180158	102703	127647	126440	53824	83411	146079	153786	90542
243011	110715	43556	304200	19812	289117	122976	87056	62443	503001	88848	17880	75257	103206	25252	18215	73389	140208	48164
6087924	5104373	4131712	5885046	4424714	4143886	3588874	2899996	3862568	4369079	4343911	5964640	4739912	3871685	5824174	5423084	6147456	5657191	4723789
423136	701092	699719	518523	528931	399870	554230	588068	554620	473849	457964	617162	654934	550923	723604	525331	640673	486613	669473
149260	118189	174868	226676	169156	179789	237732	141625	95993	147275	184529	155023	136492	164343	149790	135066	106997	179778	112410
242185	177175	117850	224356	221921	53990	134736	143742	129686	162960	194444	244525	135069	108432	180210	231266	121120	235560	64079
110070	116053	112933	103061	99090	119235	127623	116588	105472	169154	82559	93261	110148	99443	127429	57408	116976	112567	137315
103246	120325	116050	94604	86500	122069	136239	108050	104049	165118	85331	80417	111652	85161	115267	57564	121169	113480	135220
107027	123115	118771	104902	100203	125458	134339	119247	111004	174585	89022	101512	115691	109760	131145	62083	121004	120282	138891
180975	208522	198016	164241	168916	195258	208521	205527	174764	263036	139318	159235	193819	172241	211216	101525	193348	186358	213961
1796781	1793822	1698160	1716616	1741744	1918149	1933601	1979655	1796836	1864896	1753868	1785790	1833774	1867447	1909194	1769454	1896484	1877152	1744267
3671	16559	33200	29160	8353	23953	40314	11287	29633	7931	32677	34137	31717	25637	11552	10632	55128	40475	10053
51686	44693	33959	18409	41433	31966	26719	10886	33950	37337	16826	33281	32148	22925	36449	33174	35144	25844	30056
34728	55558	62404	31354	45001	29373	49519	26126	24701	19703	31421	16781	33538	31237	43501	31959	26874	59462	27774
39235	41239	43072	54326	43557	41397	39488	35163	30753	42427	41315	35124	34210	34027	43899	35866	39461	35271	44316
21990156	18011630	20863483	27068523	24343997	14620968	18559975	12554897	11832929	15173262	18730418	20370767	22064503	19545574	17073951	17035553	15049972	16409468	14367417
1001547	955355	1407171	935972	1021927	1174488	1252086	1186859	1117837	999852	1066469	1051476	1132385	991732	1109447	1099348	1134889	1039809	1169705
1273767	1158972	1890342	1275309	1374735	1416027	1579326	1401718	1392124	1189850	1349893	1317009	1421512	1250390	1365500	1365234	1357998	1271445	1435866
65496	98621	105680	127861	71003	50317	78980	81822	79557	91045	47690	75250	73268	72311	45423	117303	79900	68431	84560
2052087	1830368	2924402	1964646	2108978	2207647	2446921	2182603	2170788	1908977	2072022	2098765	2213998	1946190	2152532	2159168	2117071	2018239	2230851
21814	26971	30562	31505	13625	17129	21630	18695	22286	28043	13256	18785	25206	17395	12828	19673	33864	10496	22831
934384	791050	1301556	891618	946934	989050	1124046	968713	944761	857918	902833	936540	991154	828045	967121	961660	934380	889085	989451
1323639	1180944	1885605	1264884	1345669	1420744	1581773	1404200	1395702	1234348	1318179	1352271	1437309	1240915	1384948	1392720	1364762	1286732	1439920
1778515	1577783	2401442	1609551	1818306	1799938	2030544	1730867	1777835	1594306	1703773	1777542	1936179	1634693	1821028	1841734	1744680	1657208	1808027
655179	588182	938735	630557	673164	701277	789317	700217	696625	600467	651330	662804	715789	611529	681605	690859	680411	637787	714950
1006147	931332	1462014	965907	1038590	1100991	1233756	1095554	1070107	951302	1016651	1055347	1119565	930080	1054793	1041794	1067916	977444	1133146
381208	444260	502545	408744	446210	442959	596688	454486	450463	368569	384914	476385	373581	320046	488156	519393	797712	345981	516162
2032588	1812031	2986180	1904546	2060378	2257868	2494639	2269159	2221066	1924241	2146572	2090545	2207353	2003230	2151571	2153735	2165732	2039467	2307395
518059	568304	434917	305926	534866	275308	421877	335902	334034	391753	239355	477103	588916	245006	524930	544546	331944	276092	272534
747137	729601	965566	698572	770031	704751	779162	708195	704514	607042	664436	795020	793481	621385	730001	737563	700964	639669	744113

1332088	1234919	1936020	1226474	1348302	1420331	1584574	1398593	1416580	1234087	1332029	1379886	1451381	1283218	1407474	1392457	1416451	1282514	1472080
1791582	1597296	2458872	1671098	1755050	1843480	2042475	1834631	1859753	1620074	1765197	1789518	1900602	1673445	1789971	1866495	1788061	1699864	1859499
105921	103774	120284	71409	138972	128267	78081	99994	97462	165900	163715	82080	71746	137294	135145	137894	150218	126359	147976
79535	84828	92049	79241	78003	60723	88409	86395	79897	93655	58780	73550	77199	75966	73367	53820	76810	56472	83969
1263705	1069467	885160	1232392	914852	873461	751163	637918	827371	919448	919624	1249357	993901	824838	1213745	1153951	1302680	1190155	1000152
1359988	1248544	1964666	1265878	1392265	1454890	1618556	1452336	1451712	1250049	1379114	1424504	1494613	1304961	1414781	1421408	1427111	1304306	1499974
39498	31463	34844	32063	46118	32958	24153	30735	38331	43521	28748	26556	35332	31913	31304	34680	39317	26308	32979
54304	89917	98848	71560	80025	75665	68491	76389	85151	75473	58108	61768	76536	63067	71647	75160	56476	59086	72590
43690	61726	55831	26740	48604	58546	52312	60758	49566	62367	41992	42819	45018	58358	54457	37225	55437	42603	65329
53318	81911	98364	68615	75081	68816	58619	70435	73638	63765	57857	56517	72703	56550	71226	72637	48475	45591	65811
46419	59263	55765	33511	59116	58269	55134	54033	60061	60510	41023	36738	58897	54689	58989	46181	60755	40225	63776
99272	53712	64010	83675	98473	64308	51047	44723	41816	42682	47393	52479	50743	39234	61655	50566	77362	53830	54170
18680	16409	22225	16093	21176	17405	22355	22076	17111	17498	15983	16443	17145	21681	20300	17509	19445	20027	26972
46769	43550	42107	43463	40481	43706	34928	56267	41941	51205	43087	40593	38472	46021	34373	41333	48704	43196	40198
55872	27285	15492	57435	14679	57597	23166	17815	15470	97317	19115	7980	24306	16757	14533	17713	17025	28714	10656
84042	38406	15936	92166	9827	90806	37302	28972	24217	157292	39567	10121	29107	35122	15984	11514	22590	50547	24395
25069	35337	42605	42239	33840	37012	38616	39035	36930	24913	23266	39398	26676	27294	27137	49288	27939	30323	28960
22419	32813	35110	39452	25687	43883	41955	37198	24640	26383	18565	44473	28000	21832	26820	44484	20642	31346	26405
77121	36542	15582	92381	8593	91383	35502	33187	24103	151549	29258	7699	29995	33872	12080	11539	23577	42939	19308
49105	29897	21467	57929	15171	55635	32432	23511	17911	88919	26771	20110	24601	26281	19774	14410	25901	33387	23347
4776304	4626017	4652389	4476307	4556395	5053575	4660407	4973454	4711344	4977226	4748835	4616782	4655880	4845315	4827811	4646197	4858112	5039373	4895909
8278	4884	9938	10549	7507	8449	6910	10089	9084	6818	16775	6102	5371	8767	9260	5998	7169	9980	7580
25132	22241	20949	22507	25235	26342	24236	23197	20275	23240	19069	25462	31937	22945	22785	20927	32823	24604	21013
445772	421654	428785	413529	412460	469945	432040	466143	433497	454182	458095	429053	428779	462081	445176	431742	462669	468164	464820
3892100	3776972	3688355	3671757	3687557	4029556	3713202	3975457	3795852	4028736	3818528	3763874	3780709	3925307	3880940	3751511	3878986	4024605	3909533
1219584	1157529	1506085	1074160	1144862	1161965	1245221	1088747	1302315	1086384	1102251	1106839	1259819	1027007	1108679	1206911	1071517	1226235	1603694
87519	85936	114188	104954	55172	58881	65048	53086	80726	97897	40290	72943	90060	64933	45016	82253	105367	27426	76401
61111	51508	81070	64972	35543	58233	82119	55607	118519	71654	63723	45633	57532	68637	52589	67453	73776	47443	96786
207728	176280	194908	233827	194948	112155	203007	191190	155465	180066	287844	192132	151325	252385	166006	224671	194567	116431	178549
47097301	39219828	45221723	59666536	52351171	30043762	40968565	26414755	25557341	32511122	40960261	46163657	48601082	42539830	36738434	37321733	31384824	34675155	30312491
112989	119346	71689	39151	108586	61395	64226	62895	53270	42232	53332	93169	81445	43492	43585	63720	52168	53997	69521
123360	152927	151103	133316	143011	110949	143079	110893	120320	115074	111730	146402	167042	110032	151413	127610	136726	110942	131981
140800	116776	150516	142069	81067	104822	102847	103594	133521	136458	112033	104385	125715	114150	74585	143161	140045	66384	113324
75097	78514	94567	55239	54171	56898	64639	40837	72486	86435	46162	50844	74780	57006	59400	76960	88911	33934	54770
42894	48078	56819	43916	43616	37818	36488	32382	30203	38933	43994	36172	50439	41797	40747	41584	42822	38168	37218
44759	52781	56050	44291	46630	42010	33784	38158	34790	42153	39550	36452	46836	44073	41932	42656	39666	37213	40540
42	28	93	77	79	37	58	18	59	39	45	69	51	21	25	77	7	16	04

Overweight Persistent	Obese Occasional	Obese Persistent
66447	102628	68056
352543	799367	319011
547319	911380	489386
98147	228127	97087
518816	881823	474758
30578	56004	29335
112165	35298	96058
81229	56496	70734
32169	34447	24102
4013105	6257580	7175785
546244	870338	666612
147676	96496	93562
194923	215346	166702
102935	133388	88389
97950	119635	82677
108266	136925	90755
172366	214603	143800
1855841	1854601	1914068
9265	7784	23344
30339	32419	30151
30507	37460	49349
33075	44316	34536
14744005	15140319	15674580
1158718	1101980	1077452
1459278	1299622	1273770
77156	111625	87430
2258312	2068491	1997080
22883	15975	29142
983935	914890	880124
1448187	1323942	1272846
1848351	1693853	1625843
712390	659349	631735
1085339	1033688	984908
332271	507532	489985
2288565	2110253	2046938
306694	265350	239285
724347	673226	655145

1465556	1356900	1307027
1901765	1744445	1726917
145058	164469	140193
76459	68466	67048
860013	1340585	1520458
1498583	1365172	1326320
30175	32040	30193
65608	148502	65515
50480	62769	43235
58759	132426	58931
55751	68887	46373
55555	59189	59312
14555	18832	14107
41863	30986	43372
10783	14432	7863
12427	12248	22847
39155	48793	37988
33021	40433	44307
11313	17167	10657
17931	21608	14856
4869570	4889803	4735273
8279	5267	8751
23159	19331	19461
459632	466214	451812
3934195	3957407	3815323
1208156	1093057	1137626
76454	44973	84359
65898	48855	71361
135126	134270	128210
30653669	31790329	33592127
58040	61980	60844
124063	166463	140593
117420	98149	131508
63852	60133	69965
41492	64208	32699
44152	58644	35637
2	25	54

Table S2. Analysis of Composition Microbiomes (ANCOM) table (family level)

	Reject null hypothesis	W	0 Normal-weight	25 Normal-weight	50 Normal-weight	75 Normal-weight	100 Normal-weight	0 Obese	25 Obese	50 Obese	75 Obese	100 Obese
k__Bacteria;p__Firmicutes;c__Clostridia;o__Clostridiales;f__	True	11	92	890	1147	1872	5771	1	88	137	583	1224
k__Bacteria;p__Proteobacteria;c__Alphaproteobacteria;o__RF32;f__	True	4	1	1	105	324	1906	1	1	1	2,5	964
k__Bacteria;p__Actinobacteria;c__Coriobacteriia;o__Coriobacteriales;f__Coriobacteriaceae	True	3	9	75	200	270	1309	17	255	799	1590	5852
k__Bacteria;p__Firmicutes;c__Bacilli;o__Lactobacillales;f__Streptococcaceae	True	2	6	58	68	202	1694	28	191,5	455	869,5	4226
k__Bacteria;p__Firmicutes;c__Clostridia;o__Clostridiales;f__Peptostreptococcaceae	True	2	10	57	111	244	1280	3	94	396	584	1415
k__Bacteria;p__Firmicutes;c__Erysipelotrichi;o__Erysipelotrichales;f__Erysipelotrichaceae	True	2	81	274	379	851	2552	116	370,5	694	1067,5	5297
k__Bacteria;__;__;__;__	True	1	1	1	1	1	1	1	1	1	1	468
k__Bacteria;p__Cyanobacteria;c__Chloroplast;o__Streptophyta;f__	True	1	1	1	1	1	5	1	1	1	1	88
k__Bacteria;p__Firmicutes;c__Bacilli;o__Gemellales;f__Gemellaceae	True	1	1	1	1	1	15	1	1	1	1	30
k__Bacteria;p__Firmicutes;c__Clostridia;o__Clostridiales;f__Peptococcaceae	True	1	1	1	1	30	278	1	1	1	1	20
k__Bacteria;p__Firmicutes;c__Clostridia;o__Clostridiales;f__Ruminococcaceae	True	1	5462	11557	18977	23544	35537	5306	13660	17433	25886,5	46587
k__Bacteria;p__Firmicutes;c__Clostridia;o__Clostridiales;f__[Tissierellaceae]	True	1	1	1	1	1	108	1	1	7	11,5	416
k__Bacteria;p__Tenericutes;c__Mollicutes;o__RF39;f__	True	1	1	1	1	1	30	1	1	1	1	195
k__Archaea;p__Euryarchaeota;c__Methanobacteria;o__Methanobacteriales;f__Methanobacteriaceae	True	0	1	1	1	1	93	1	1	1	1	202
k__Bacteria;p__Actinobacteria;c__Actinobacteria;o__Bifidobacteriales;f__Bifidobacteriaceae	True	0	21	155	277	1097	8457	1	412	1723	3415	16556
k__Bacteria;p__Bacteroidetes;c__Bacteroidia;o__Bacteroidales;__	True	0	1	1	1	1	1	1	1	1	1	2401
k__Bacteria;p__Bacteroidetes;c__Bacteroidia;o__Bacteroidales;f__	True	0	1	1	1	1	1	1	1	1	1	1392

k__Bacteria;p__Bacteroidetes;c__Bacteroidia;o__Bacteroidales;f__Bacteroidaceae	True	0	1217	13062	24483	35934	69651	301	11864	17099	31761,5	56990
k__Bacteria;p__Bacteroidetes;c__Bacteroidia;o__Bacteroidales;f__Porphyromonadaceae	True	0	647	1024	1523	3240	7381	1	1675	2869	4587	9601
k__Bacteria;p__Bacteroidetes;c__Bacteroidia;o__Bacteroidales;f__Prevotellaceae	True	0	1	8	14	4601	29116	1	28,5	2047	12558	28109
k__Bacteria;p__Bacteroidetes;c__Bacteroidia;o__Bacteroidales;f__Rikenellaceae	True	0	282	3485	6666	8183	24931	14	1792,5	5504	9671,5	18409
k__Bacteria;p__Bacteroidetes;c__Bacteroidia;o__Bacteroidales;f__S24-7	True	0	1	1	1	8	3935	1	1	1	1	4958
k__Bacteria;p__Bacteroidetes;c__Bacteroidia;o__Bacteroidales;f__[Barnesiellaceae]	True	0	12	1146	2256	3262	4411	10	820	2031	3387	8252
k__Bacteria;p__Bacteroidetes;c__Bacteroidia;o__Bacteroidales;f__[Odoribacteraceae]	True	0	84	706	1006	1881	4647	1	316,5	537	1133,5	4029
k__Bacteria;p__Bacteroidetes;c__Bacteroidia;o__Bacteroidales;f__[Paraprevotellaceae]	True	0	1	1	1	105	1567	1	1	1	413	2234
k__Bacteria;p__Cyanobacteria;c__4C0d-2;o__YS2;f__	True	0	1	1	1	206	2122	1	1	1	1	1100
k__Bacteria;p__Firmicutes;c__Bacilli;o__Lactobacillales;f__Carnobacteriaceae	True	0	1	1	1	3	18	1	1	1	8,5	19
k__Bacteria;p__Firmicutes;c__Bacilli;o__Lactobacillales;f__Enterococcaceae	True	0	1	1	1	1	25	1	1	1	1	51
k__Bacteria;p__Firmicutes;c__Bacilli;o__Lactobacillales;f__Lactobacillaceae	True	0	1	1	1	1	725	1	1	1	40	832
k__Bacteria;p__Firmicutes;c__Bacilli;o__Turicibacterales;f__Turicibacteraceae	True	0	1	1	14	31	309	1	1	5	36	241
k__Bacteria;p__Firmicutes;c__Clostridia;o__Clostridiales;__	True	0	1	52	155	640	1038	1	10	72	201,5	733
k__Bacteria;p__Firmicutes;c__Clostridia;o__Clostridiales;f__Christensenellaceae	True	0	1	16	38	720	5802	1	2	26	68,5	2883
k__Bacteria;p__Firmicutes;c__Clostridia;o__Clostridiales;f__Clostridiaceae	True	0	11	248	365	887	7774	1	87,5	282	456	1608
k__Bacteria;p__Firmicutes;c__Clostridia;o__Clostridiales;f__Dehalobacteriaceae	True	0	1	1	1	13	65	1	1	1	1	24
k__Bacteria;p__Firmicutes;c__Clostridia;o__Clostridiales;f__EtOH8	True	0	1	1	1	1	118	1	1	1	1	1
k__Bacteria;p__Firmicutes;c__Clostridia;o__Clostridiales;f__Lachnospiraceae	True	0	3147	7875	11194	20005	37066	4847	10391	15206	19156,5	43168
k__Bacteria;p__Firmicutes;c__Clostridia;o__Clostridiales;f__Veillonellaceae	True	0	2046	2716	4729	6734	25066	205	3791,5	8057	15764	154322

k__Bacteria;p__Firmicutes;c__Clostridia;o__Clostridiales;f__[Mogibacteriaceae]	True	0	10	39	87	124	208	1	37	118	165,5	431
k__Bacteria;p__Firmicutes;c__Clostridia;o__SHA-98;f__	True	0	1	1	1	20	60	1	1	1	1	22
k__Bacteria;p__Fusobacteria;c__Fusobacteriia;o__Fusobacteriales;f__Fusobacteriaceae	True	0	1	1	1	1	10	1	1	1	1	4700
k__Bacteria;p__Lentisphaerae;c__[Lentisphaeria];o__Victivallales;f__Victivallaceae	True	0	1	1	1	8	69	1	1	1	1	220
k__Bacteria;p__Proteobacteria;c__Alphaproteobacteria;__;__	True	0	1	1	1	1	999	1	1	1	1	1
k__Bacteria;p__Proteobacteria;c__Alphaproteobacteria;o__Rhodospirillales;f__Acetobacteraceae	True	0	1	1	1	1	1	1	1	1	1	80
k__Bacteria;p__Proteobacteria;c__Betaproteobacteria;o__Burkholderiales;f__Alcaligenaceae	True	0	181	1584	1885	5177	8536	129	413,5	1039	4132	11418
k__Bacteria;p__Proteobacteria;c__Deltaproteobacteria;o__Desulfovibrionales;f__Desulfovibrionaceae	True	0	1	54	274	352	640	1	21	235	404,5	3039
k__Bacteria;p__Proteobacteria;c__Epsilonproteobacteria;o__Campylobacterales;f__Campylobacteraceae	True	0	1	1	1	1	81	1	1	4	9,5	292
k__Bacteria;p__Proteobacteria;c__Gammaproteobacteria;o__Aeromonadales;f__Succinivibrionaceae	True	0	1	1	1	1	113	1	1	1	1	4685
k__Bacteria;p__Proteobacteria;c__Gammaproteobacteria;o__Enterobacteriales;f__Enterobacteriaceae	True	0	1	25	386	819	2886	1	36	141	946	4940
k__Bacteria;p__Proteobacteria;c__Gammaproteobacteria;o__Pasteurellales;f__Pasteurellaceae	True	0	1	9	25	199	1734	1	25,5	86	299	1536
k__Bacteria;p__Proteobacteria;c__Gammaproteobacteria;o__Pseudomonadales;f__Pseudomonadaceae	True	0	1	1	1	1	1	1	1	1	1	341
k__Bacteria;p__Spirochaetes;c__[Brachyspirae];o__[Brachyspirales];f__Brachyspiraceae	True	0	1	1	1	1	1	1	1	1	1	145
k__Bacteria;p__TM7;c__TM7-3;o__;f__	True	0	1	1	1	16	26	1	1	1	14,5	399
k__Bacteria;p__TM7;c__TM7-3;o__CW040;f__	True	0	1	1	1	1	18	1	1	1	1	122
k__Bacteria;p__Tenericutes;c__Mollicutes;o__Mycoplasmatales;f__Mycoplasmataceae	True	0	1	1	1	1	1	1	1	1	1	153
k__Bacteria;p__Tenericutes;c__RF3;o__ML615J-28;f__	True	0	1	1	1	1	1597	1	1	1	1	1

k__Bacteria;p__Verrucomicrobia;c__Opitutae;o__[Cerasicoccales];f__[Cerasicoccaceae]	True	0	1	1	1	1	89	1	1	1	1	16
k__Bacteria;p__Verrucomicrobia;c__Verrucomicrobiae;o__Verrucomicrobiales;f__Verrucomicrobiaceae	True	0	1	18	717	5539	9604	1	6	21	402	14420
